# Combination of bioaffinity ultrafiltration-UFLC-ESI-Q/TOF-MS/MS, *in silico* docking and multiple complex networks to explore antitumor mechanism of topoisomerase I inhibitors from *Artemisiae Scopariae* Herba

**DOI:** 10.1186/s12906-023-04146-x

**Published:** 2023-09-12

**Authors:** Tong Chen, Jingbo Hu, Huan Wang, Nana Tan, Jianzhao Qi, Xiaoling Wang, Le Wang

**Affiliations:** 1 Shaanxi Key Laboratory of Phytochemistry, College of Chemistry and Chemical Engineering, Baoji University of Arts and Sciences, High-tech Avenue 1#, Baoji, 721013 China; 2 College of Electronic and Electrical Engineering, Baoji University of Arts and Sciences, Baoji, 721013 China; 3 College of Computer Science and Technology, Baoji University of Arts and Sciences, Baoji, 721013 China; 4 Shaanxi Key Laboratory of Natural Products & Chemical Biology, College of Chemistry & Pharmacy, Northwest A & F University, Yangling, 712100 China

**Keywords:** *Artemisiae Scopariae* Herba, Topoisomerase I inhibitors, LC-MS, *In silico* docking, Multiple complex networks

## Abstract

**Background:**

*Artemisiae Scopariae* Herba (ASH) has been widely used as plant medicine in East Asia with remarkable antitumor activity. However, the underlying mechanisms have not been fully elucidated.

**Methods:**

This study aimed to construct a multi-disciplinary approach to screen topoisomerase I (topo I) inhibitors from ASH extract, and explore the antitumor mechanisms. Bioaffinity ultrafiltration-UFLC-ESI-Q/TOF-MS/MS was used to identify chemical constitution of ASH extract as well as the topo I inhibitors, and *in silico* docking coupled with multiple complex networks was applied to interpret the molecular mechanisms.

**Results:**

Crude ASH extract exhibited toxicogenetic and antiproliferative activities on A549 cells. A series of 34 ingredients were identified from the extract, and 6 compounds were screened as potential topo I inhibitors. Docking results showed that the formation of hydrogen bond and π-π stacking contributed most to their binding with topo I. Interrelationships among the 6 compounds, related targets and pathways were analyzed by multiple complex networks model. These networks displayed power-law degree distribution and small-world property. Statistical analysis indicated that isorhamnetin and quercetin were main active ingredients, and that chemical carcinogenesis-reactive oxygen species was the critical pathway. Electrophoretic results showed a therapeutic effect of ASH extract on the conversion of supercoiled DNA to relaxed forms, as well as potential synergistic effect of isorhamnetin and quercetin.

**Conclusions:**

The results improved current understanding of *Artemisiae Scopariae* Herba on the treatment of tumor. Moreover, the combination of multi-disciplinary methods provided a new strategy for the study of bioactive constituents in medicinal plants.

**Supplementary Information:**

The online version contains supplementary material available at 10.1186/s12906-023-04146-x.

## Background

*Artemisiae Scopariae* Herba (ASH) is the dried aerial part of *Artemisia scoparia* Waldst.et Kit [1], which is widespread across Eurasia from central Europe to Japan and China. It is one of the most commonly used Traditional Chinese Medicine (TCM) herbal, and first recorded as medicinal plant about two thousand years ago in Shen Nong Ben Cao Jing, the earliest pharmacology monograph of TCM [2]. Chemical constituents of ASH are complicated, containing coumarous, flavonoids, organic acids, phenolic acids, and terpenoids [3]. This plant has diverse bioactivities, including neuroprotection, antivirus, analgesic, hypotensive, hepatoprotective and anti-osteoporotic effects [4]. Pharmacological researches showed distinct antitumor activity of ASH [5]. Its extract could significantly suppress the growth and colony formation of hepatocellular carcinoma (HCC) cells [6], as well as HS578T cells [7], which were epithelial cells isolated from human breast cancer tissues. The ethyl acetate part of ASH was reported to inhibit growth and migration of HCC cells by changing mitochondrial membrane potential [8]. Several naturally occurring components of ASH also possessed obvious antitumor activities. Capillarisin in ASH was reported to exhibit a certain inhibitory effect on prostate cancer cells, and arrest the growth of cancer cells through the IL-6/STAT3 pathway [9]. In this pathway, interleukin-6 (IL-6) rapidly activates the signal transducer and activator of transcription 3 (STAT3), which is a major mediator regulating signal transduction from IL-6 to the nucleus and inducing the transcription of proliferation associated genes. These reports suggested great potential of ASH in the treatment of tumors, whereas the action mechanism was complex.

Topoisomerase I (topo I), an enzyme involved in the relaxation of supercoiled DNA, is an important biological target in the treatment of tumor [10, 11]. Inhibitors of topo I could limit activity of the enzyme in its enzymatic cycle [12]. Natural product has become an active field of research for topo I inhibitors as their low cytotoxicity [13]. A collection of low cytotoxic or non-cytotoxic compounds with various structures were identified from plants and their symbiotic organisms [14]. For instance, camptothecin and its synthetic derivatives show good inhibitory activity of topo I [15]. The 9-methoxycamptothecin from *Nothapodytes nimmoniana (J. Graham) Mabberl*y exhibited significant antitumor activity in vitro as a topo I inhibitor [16]. Some of its derivatives have been approved for clinical use in tumor therapy, such as irinotecan, topotecan, and belotecan [17]. Additionally, many flavonoids were found to be topo I inhibitors, including morin, fisetin, quercetin, and myricetin [18]. Structural features of active site and action of various topo I inhibitors also become a hot topic [19]. Curcumin and its natural derivatives, cyclocurcumin and curcumin sulphate were predicted to be the most potent topo I inhibitors, docked at the site of DNA cleavage parallel to the axis of DNA base pairing, and that residues Arg364, Asn722 and base A113 played an important role [20]. However, very few investigations concerned with topo I inhibitors from ASH and their mechanisms.

The underlying mechanisms of compounds from medicinal plants against tumor are obscured as complex chemical composition and diverse pathways [21]. Recently, complex network has emerged as an effective solution of information mining on big data, and widely applied in many fields about natural science and social science [22–24]. It has been proved to be an excellent tool in the researches of complex diseases including cancer, AIDS and asthma [25, 26]. This methodology has also been used in the studies of medicines and natural products [24, 27]. For instance, Jia et al.(2021) reported a complex network of prescribed herbs of traditional Chinese medicine in the treatment of hypertensive nephropathy, and that fourteen herbs were found to act through down-regulating the expression of inflammatory cytokines such as TNF, IL-1B, and IL-6 and the NF-κB and MAPK signaling pathways [28]. Dong et al. (2021) applied this method to investigate the mechanism of the *Astragalus membranaceous* and *Angelica sinensis* in the treatment of diabetic nephropathy (DN), which might treat DN by acting on vascular endothelial growth factor A (VEGFA), tumor Protein P53 (TP53), interleukin-6 (IL-6), tumor necrosis factor (TNF), microtubule affinity regulating kinase 1 (MARK1), etc., and regulate apoptosis, oxidative stress, inflammation, glucose, and lipid metabolism processes [29]. These reports suggested that complex network could be used to explore the antitumor mechanism of topo I inhibitors from ASH from the system point of view.

In the present study, an interdisciplinary analysis was performed to screen topo I inhibitors from ASH extract, and explore the underlying mechanisms. The topo I inhibitors were identified by bioaffinity ultrafiltration-UFLC-ESI-Q/TOF-MS/MS. Combination mechanisms of hydroxygenkwanin, chlorogenic acid, and topo I were revealed using *in silico* docking. Multiple complex networks were constructed based on the selected topo I inhibitors, related target proteins and pathways. Characteristics of the networks were further analyzed, designed to clarify the antitumor mechanisms. This study would provide a new strategy for the research of topo I inhibitors originated from medicinal plants.

## Methods

### Materials and preparation of samples

The above-ground part of crude *Artemisiae Scopariae* Herba was obtained from Baoji Chenguang Biotechnology Co., Ltd (Baoji, China). Samples were collected at the seedling stage. Plant species was authenticated by Prof. Shifeng Ni from Northwest University, Key Laboratory of Resource Biology and Biotechnology in Western China, Northwest University, Xi’an, China. A voucher specimen (BJWLXY-CC-SKLP220301) was deposited at Shaanxi Key Laboratory of Phytochemistry, Baoji University of Art and Sciences, Baoji, China. Dried ASH powders (100 g), consisted of leaf and stem of the plant, were treated with ultrasonic-assisted extraction method using 50% ethanol for 30 min, and repeated for another two times. Then, the 50% ethanol solution was leached with ethyl acetate (saturated by water). Solvents were subsequently removed by a vacuum centrifugal concentrator. The residues were stored at -80℃ for 2 weeks before use.

### Chemicals and materials

DNA topoisomerase I (Topo I, human) and sulphorhodamine B (SRB) were acquired from Sigma-Aldrich (St Louis, MO, USA). Amicon Ultra-0.5 centrifugal filters (3 kDa) and ultrapure water were obtained from Millipore Co. Ltd. (Bedford, MA, USA). Methanol and Acetonitrile (HPLC grade) were purchased from Merck (Darmstadt, Germany). Other chemicals were analytical grade and supplied by Aladdin industrial corporation (Shanghai, China). Fetal bovine serum (FBS), plasmid pBR322, 4 S Green Plus nucleic acid dye and TAE buffer were obtained from Sangon Biotech Co., Ltd. (Shanghai, China). A549 cells were supplied by Beyotime Institute of Biotechnology (Haimen, China). DNA topoisomerase I (Topo I, human) was acquired from Sigma-Aldrich (St Louis, MO, USA).

### Cell culture, cell viability, and morphological assessment

The A549 cells (epithelial cells isolated from human lung cancer tissues) were maintained in Dulbecco’s modified eagle medium (DMEM) supplemented with 10% fetal bovine serum (FBS, *v*/*v*), glutamine (2 mM), and antibiotics (penicillin and streptomycin, 100 units/mL) in a 5% CO_2_ atmosphere at 37 °C. Cells were seeded overnight in a 96-well plate (6 × 10^3^ cells/well), and treated with ASH extract, quercetin (positive control) and dimethyl sulfoxide (DMSO, negative control) for 48 h. SRB assay was then performed to measure cell viability according to previous report [30]. Absorbance at 530 nm was recorded using a microplate reader (Synergy NEO 2, Bio-Tek). IC_50_ was acquired by modified Karber’s method [31]. Subsequently, Hoechst 33,342 staining was applied to detect the apoptotic morphology. A549 cells were cultured in 12-well plates to adhere for 24 h, and treated with ASH extract or DMSO (control) for 24 h. After incubation, Hoechst 33,342 was added to the cells. The stained cells were observed using a fluorescent microscopy (ECLIPSE Ti-E, Nikon).

### Bioaffinity ultrafiltration for screening potential topo I inhibitors

ASH extract (20 µL, 10 mg/mL) were incubated with topo I (20 µL, 12.5 U) and 460 µL buffer (pH 7.9) at 37 °C for 30 min. Then the samples were transferred into ultrafiltration tubes (3 kDa) and centrifuged at 14,000 g for 20 min. The tubes were flushed using 500 µL buffer for two times to remove the compounds not bound to the enzyme. Subsequently, methanol (500 µL, 50%) was added to the samples and incubated for 10 min, then centrifuged at 14,000 g for 20 min, which also repeated twice. The filtrate were combined and processed with 0.22 μm filter membrane.

### UFLC-ESI-Q/TOF-MS/MS analysis

The samples were analyzed by a LC-20AD^XR^ UFLC-4600 Q/TOF system (Shimadzu, Japan & AB SCIEX, USA), equipped with a Shim-pack XR-ODS column (100 mm×2.0 mm, 2.2 μm; Shimadzu, Japan). Detection parameters were set according to our previous report with minor modifications [32]. The injection volume was 1 µL, and that flow rate was 0.25 mL/min. The mobile phase included water (A) and methanol (B) containing formic acid (0.1%). Gradient elution program was set as follow: 10% B at 0-0.5 min, 10–30% B at 0.5-2 min, 30–48% B at 2–15 min, 48–100% B at 15–20 min, 100% B at 20–23 min. Mass scan range were 100–1000 *m*/*z*. Data were collected by Analyst (ver. 1.7, AB SCIEX, USA), and processed with PeakView (ver. 2.2, AB SCIEX, Canada) and MasterView (ver. 1.1, AB SCIEX, Canada). Compounds were identified by Natural Products HR-MSMS Spectral Library (ver. 1.0.1, AB SCIEX, USA). Mass tolerance of the accurate molecular weight was set as ± 5 ppm. Furthermore, MS/MS fragment patterns were analyzed to verify the results.

### *In Silico* docking

Structures of potential topo I inhibitors from ASH were saved in Mol format. PDB code of Human DNA Topoisomerase I was 1T8I. Docking was performed by Discovery Studio (BIOVIA). Original ligands and water were removed from the complex, and that hydrogen atoms were added. Proteins were refined with CHARMm force field. Active binding sites were Arg 488, Lys 532, Arg 590, His 632, and Tyr 723 according to the literature [33]. Pose cluster radius was set at 0.5 and the other parameters were at the default values. Scores of -CDOCKER interaction energy were investigated, and that camptothecin was used as control.

### Construction and analysis of multiple complex networks

To explore the underlying mechanisms, multiple complex networks were build based on the selected topo I inhibitors from ASH, their target proteins and involved pathways. Targets were predicted by Similarity ensemble approach (SEA) (https://sea.bkslab.org/) [34], on basis of set-wise chemical similarity among proteins and their ligands. UniProt IDs of these proteins were collected for information standardization. The involved pathways were analyzed by DAVID (https://david.ncifcrf.gov/, *P* < 0.05). Multiple complex networks were then built, consisted of large amounts of nodes and edges. Nodes represent the topo I inhibitors, related target proteins or involved pathways, and that edges exhibit interactions between them. The networks were visualized using Pajek (ver. 5.16, by Andrej Mrvar and Vladimir Batagelj).

First, an ingredient-pathway interaction (IPI) network was constructed. If targets of an inhibitor node *i* were involved in pathway node *j*, there is a connection between *i* and *j*. Besides, if two inhibitors nodes *i*_1_, *i*_2_ had common targets, or a target is both involved in pathway nodes *j*_1_, *j*_2_, a connection emerges between them. Then, three subnetworks were extracted from IPI network for different interpretations, including the ingredient-ingredient interaction (TTI) network, ingredient-pathway bimodal (IPB) network, and pathway-pathway interaction (PPI) network. TTI network describes interactions between inhibitors connected with identical targets. IPB network exhibits connections between the inhibitors and pathways possessing common targets. Besides, the PPI network characterizes relationships between pathways involved identical targets.

The network characteristics were calculated to screen critical nodes, using MATLAB 2016a (The MathWorks Inc.). Most parameters have been described in our previous work [35], including degree (*k*), the average degree *< k>*, degree distribution *P(k)*, diameter (*D*), average path length (*L*), clustering coefficient (*C*), and three centrality indicators, degree centrality (*C*_*d*_), betweenness centrality (*C*_*b*_), and closeness centrality (*C*_*c*_). Part of these parameters and their formulas were listed in Table 2.

### Topo I inhibitory binding assay

Inhibition of topo I for ASH extract and the selected ingredients was tested by agarose gel electrophoresis. The reaction mixtures contained plasmid pBR322 (0.175 µg), the samples (dissolved in DMSO), topo I (0.5 U), diluted Topo I buffer (6×, 2 µL), BSA (0.1%, 2 µL). Camptothecin was applied as positive control. The mixtures were incubated at 37 °C for half an hour. Electrophoresis was conducted on 1% agarose gel (150 V, 30 min). The gels were dyed in 4 S Green Plus nucleic acid stain (10,000X) for 30 min and discolored for another 30 min, then visualized using a G:BOX Chemi XRQ gel imaging system (Syngene, Germany).

## Results

### Antitumor effect

**Fig. 1:**
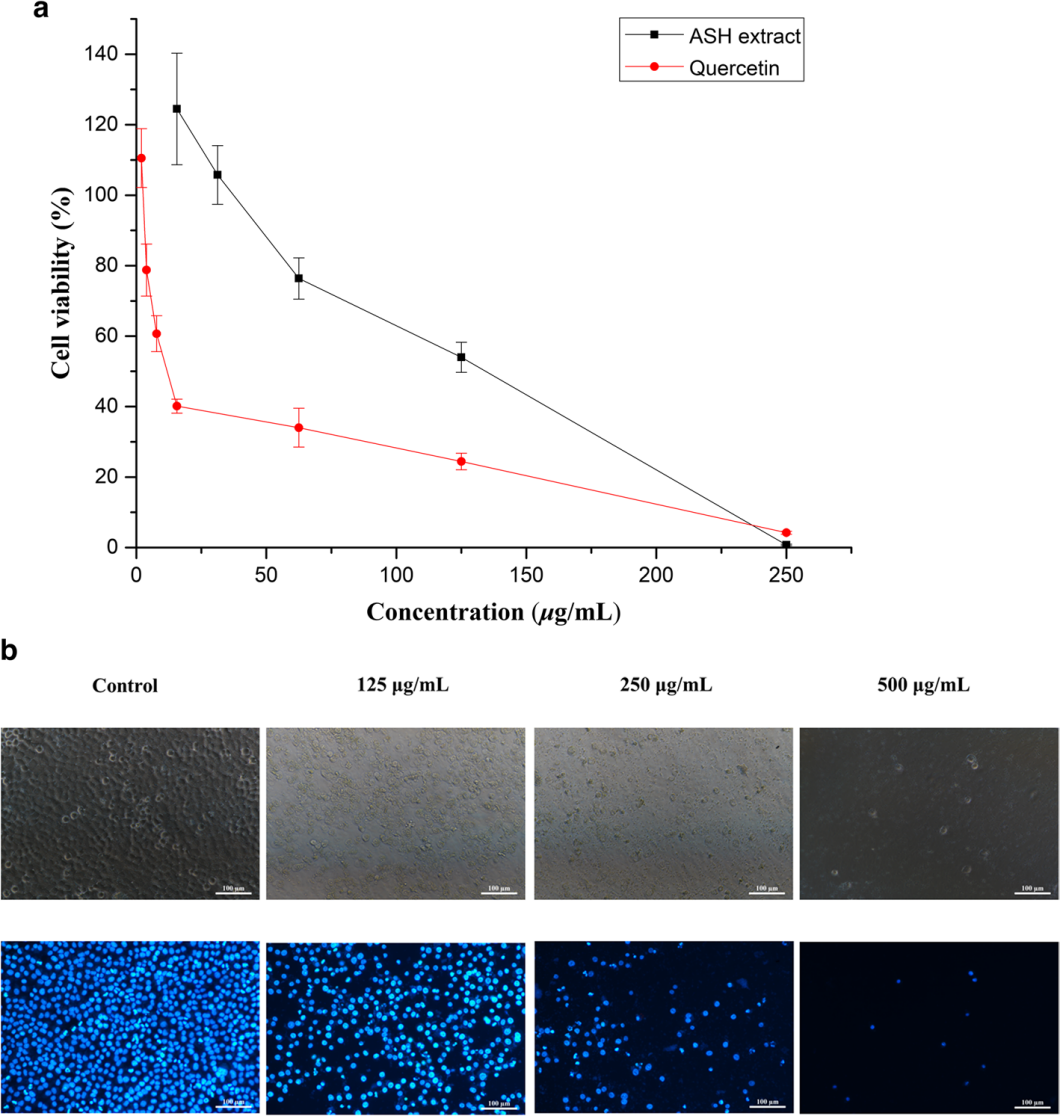
ASH extract promotes viability deficiency and apoptosis of A549 cells. **a**, Cytotoxicity of ASH extract to A549 cells by SRB assay. Cells were treated with ASH extract for 48 h, and that quercetin was used as positive control. **b**, Nuclear morphological changes by Hoechst 33,342 staining. The phase contrast (top) and fluorescence (bottom) images were collected using fluorescence microscopy

Antitumor effect of ASH extract was investigated on A549 cell. The cell viability decreased remarkably with increasing concentration of the sample (Fig. 1a). Finally, the crude plant extract exhibited a potential antitumor effect (IC_50_ = 113.64 µg/mL). Morphological assay of cell death was performed using Hoechst 33,342 staining for fluorescence microscopy. A549 cells were exposed to gradient concentrations of ASH extract for 24 h. The nuclear was slightly condensed at 125 µg/mL ASH extract, whereas untreated controls showed evenly dispersed blue fluorescence. Moreover, lots of chromatin condensation or dense stained debris was found with increasing concentration of the sample (Fig. 1b). These results indicated that the ASH extract could induce apoptotic morphology on A549 cells.

### Chemical composition of ASH extract and topoisomerase I inhibitors

**Fig. 2:**
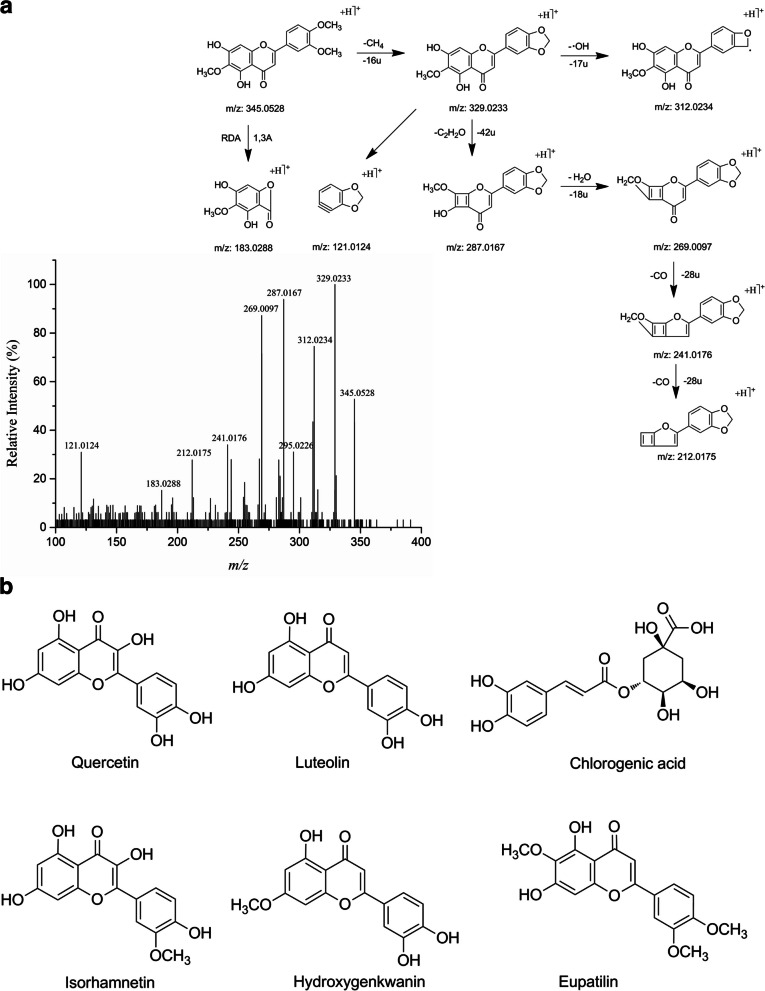
Potential topo I inhibitors identified from ASH extract. **a**, ESI-MS/MS spectrum and fragmentation pathways of eupatilin. **b**, Chemical structures of 6 potential topo I inhibitors from ASH extract

Chemical composition of ASH extract was investigated using UFLC-ESI-Q/TOF-MS/MS. A total of 34 compounds were identified. These ingredients contained 3 coumarins, 10 flavones, 4 organic acids, 5 alkaloids, 4 terpenes, and other compounds (Table 1). Fragmentation analysis was performed on these ingredients for further confirmation. For instance, compound 11 has a [M + H]^+^ peak at m/z 345.0528 (Fig. 2a). The MS/MS spectra shows an adjacent methoxylated flavone characteristic loss of CH_4_ (16 Da), generating a product ion of m/z 329.0233. Furthermore, the ion m/z 329.0233 produces fragment ions at m/z 121.0124, 287.0167 and 312.0234. The product ion m/z 287.0167 derives from loss of C_2_H_2_O (42 Da). It also loses a molecule of H_2_O (18 Da) to produces a fragment at m/z 269.0097. Subsequently, one or two neutral loss of CO (28 Da) from m/z 269.0097 produces ions at m/z 241.0176 and m/z 212.0175. In addition, the production ion at m/z 183.0288 is attributed to ^1,3^ A^+^, and that substituent groups of two OH and an OCH_3_ are located in A-ring. Therefore, compound 11 was finally identified as eupatilin, which was also supported by previous report [36].

**Table 1:** Ingredients identified from *Artemisiae Scopariae* Herba extract by UFLC-ESI-Q/TOF-MS/MS

No.	Ingredients	Category	*t*_R_ (min)	Formula	CAS Number	Molecular Weight	Measured m/z^a^	Error^b^ (ppm)
1	6-Hydroxy-7,8-dimethoxycoumarin	Coumarin	4.16	C_11_H_10_O_5_	107078-01-3	222.0528	223.0600	-0.4
2	Isoscopoletin	Coumarin	4.15	C_10_H_8_O_4_	776-86-3	192.0423	193.0497	0.8
3	6,7-Dimethoxycoumarin	Coumarin	4.37	C_11_H_10_O_4_	120-08-1	206.0579	207.0650	-0.8
4	Kaempferol-3-O-rutinoside	Flavone	3.82	C_27_H_30_O_15_	17650-84-9	594.1584	595.1653	-0.8
5	Quercetin *	Flavone	4.34	C_15_H_10_O_7_	117-39-5	302.0427	303.0498	-0.5
6	Luteolin *	Flavone	4.53	C_15_H_10_O_6_	491-70-3	286.0477	287.0549	-0.3
7	Scutellarein	Flavone	4.53	C_15_H_10_O_6_	529-53-3	286.0477	287.0549	-0.3
8	Isorhamnetin *	Flavone	4.58	C_16_H_12_O_7_	480-19-3	316.0583	317.0657	0.4
9	5,7,3’-Trihydroxy-6,4’,5’-trimethoxyflavone	Flavone	5.91	C_18_H_16_O_8_	78417-26-2	360.0845	361.0919	0.3
10	Hydroxygenkwanin *	Flavone	6.09	C_16_H_12_O_6_	20243-59-8	300.0634	301.0707	0.1
11	Eupatilin *	Flavone	6.52	C_18_H_16_O_7_	22368-21-4	344.0896	345.0969	0.0
12	Genkwanin	Flavone	7.18	C_16_H_12_O_5_	437-64-9	284.0685	285.0758	0.1
13	Irisflorentin	Flavone	7.30	C_20_H_18_O_8_	41743-73-1	386.1002	387.1077	0.8
14	Phenylalanine	Organic Acid	2.84	C_9_H_11_NO_2_	63-91-2	165.0790	166.0864	0.9
15	8-Epiloganic acid	Organic Acid	3.91	C_16_H_24_O_10_	82509-41-9	376.1369	377.1445	0.7
16	Chlorogenic acid *	Organic Acid	5.23	C_16_H_18_O_9_	327-97-9	354.0951	355.1022	-0.3
17	Linolenic acid	Organic Acid	10.70	C_18_H_30_O_2_	463-40-1	278.2246	279.2319	0.1
18	*α*-Asarone	Phenylpropanoid	4.81	C_12_H_16_O_3_	2883-98-9	208.1099	209.1174	1.0
19	Salidroide + NH_3_	Phenyl glycoside	3.45	C_14_H_20_O_7_.NH_3_	10338-51-9	317.1475	318.1546	-0.4
20	Sedanolide	Phenphthalide	7.23	C_12_H_18_O_2_	6415-59-4	194.1307	195.1381	0.8
21	Aurantio-obtusin	Anthraquinone	6.01	C_17_H_14_O_7_	67979-25-3	330.0739	331.0809	-0.8
22	Ethyl vanillin	Phenol	3.59	C_9_H_10_O_3_	121-32-4	166.0630	167.0703	0.5
23	Butylparaben	Phenol	4.88	C_11_H_14_O_3_	94-26-8	194.0943	195.1018	1.0
24	6-Shogaol	Phenol	7.68	C_17_H_24_O_3_	555-66-8	276.1725	277.1799	0.4
25	Genipin	Terpene	3.84	C_11_H_14_O_5_	6902-77-8	226.0841	227.0915	0.3
26	Curcumenol	Terpene	7.10	C_15_H_22_O_2_	19431-84-6	234.1620	235.1692	-0.2
27	Linderane	Terpene	8.12	C_15_H_16_O_4_	13476-25-0	260.1049	261.1124	1.0
28	Patchouli alcohol (loss H_2_O)	Terpene	8.21	C_15_H_24_	5986-55-0	204.1878	205.1950	-0.3
29	Anise oil	Volatile oil	4.81	C_10_H_12_O	4180-23-8	148.0888	149.0962	0.5
30	Vitamin B6	Alkaloid	2.57	C_8_H_11_NO_3_	65-23-6	169.0739	170.0812	0.2
31	Nicotinamide	Alkaloid	2.73	C_6_H_6_N_2_O	98-92-0	122.0480	123.0554	0.8
32	Cordycepin	Alkaloid	3.20	C_10_H_13_N_5_O_3_	73-03-0	251.1018	252.1089	-0.9
33	Bicuculline	Alkaloid	3.71	C_20_H_17_NO_6_	485-49-4	367.1056	368.1125	-1.0
34	Piperine	Alkaloid	4.69	C_17_H_19_NO_3_	94-62-2	285.1365	286.1439	0.4

^a^Measured m/z of peak [M + H]^+^

^b^Mass accuracy between the calculated m/z and measured m/z of peak [M + H]^+^; *, potential topo I inhibitors from *Artemisiae Scopariae* Herba extract

Bioaffinity ultrafiltration could facilitate the separation of ligand-receptor [37, 38]. Topoisomerase I inhibitors were screened from ASH extract by ultrafiltration, and also identified using UFLC-ESI-Q/TOF-MS/MS. Samples were first incubated with topo I, and the unbound compounds were separated. Subsequently, the ligands were dissociated and filtered out. A total of 6 compounds showed larger peak areas in sample group than those in the control group, including eupatilin (11), isorhamnetin (8), luteolin (6), quercetin (5), chlorogenic acid (16), and hydroxygenkwanin (10). Their molecular structures were displayed in Fig. 2b. These ingredients were considered as potential topo I inhibitors.

### Binding mechanism

**Fig. 3:**
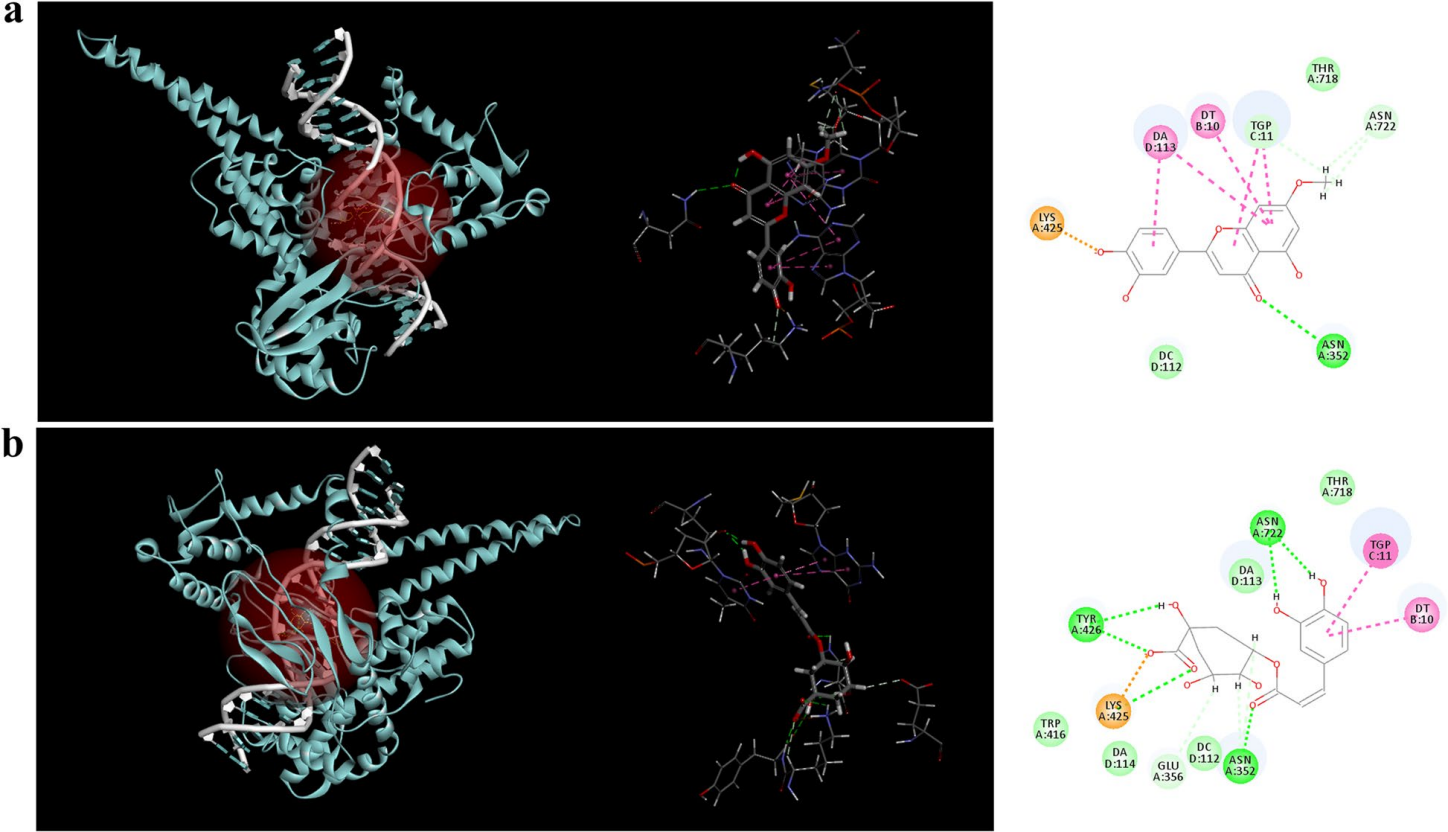
Binding profiles of topo I inhibitors from ASH extract. PDB ID of topo I is 1T8I. **a**, Interactions of hydroxygenkwanin and topo I. **b**, Interactions of chlorogenic acid and topo I. Light pink line indicates π-alkyl ineraction. Dark pink line indicates π-π stacked. Orange line indicates π-anion interaction. Light green line indicates carbon hydrogen bond. Green line indicates van der Waals force. Dark green line indicates conventional hydrogen bond

Differences in functional groups of natural products and stereochemical conformation have significant influence on topo I inhibition [18]. In this part, *in silico* docking was conducted among the 6 inhibitors selected above and topo I to verify the ultrafiltration results, and investigate detailed binding sites and orientations. The results indicated that the active sites contained Arg 364, Asp 533, Asn 722, and several important residues, nucleotides DC112, DA113, and DT10. CDOCKER interaction energy of the 6 inhibitors and topo I ranged from 50 to 75 kcal·mol^−1^ (Additional file 1), which were all higher than that of the control camptothecin (37.76 kcal·mol^−1^). It proved that all the 6 inhibitors from ASH extract were tightly connected with topo I. Their specific binding mechanisms were further analyzed in detail. Hydroxygenkwanin and chlorogenic acid were selected as examples. As shown in Fig. 3a, hydroxygenkwanin was bound to the active pocket of topo I through the formation of three carbon hydrogen bonds with residues Tgp 11 and Asn 722, as well as another hydrogen bond with Asn 352. Besides, π-alkyl, π-π stacking and π-anion existed between this ligand and other residues or bases of topo I. Figure 3b illustrated that chlorogenic acid was bound to topo I through the formation of three carbon hydrogen bonds with the residue Asn 352, Glu 356, and six conventional hydrogen bonds with Asn 352, Tyr 426, Asn 722 and Lys 425. Furthermore, it also interacted with topo I through π-π stacking, and van der Waals forces with other residues and bases. These results demonstrated that the formation of hydrogen bond and π-π stacking contributed most to the binding of the selected inhibitors and topo I.

### Multiple complex networks and key elements

#### Ingredient-Pathway Interaction (IPI) Network

**Fig. 4:**
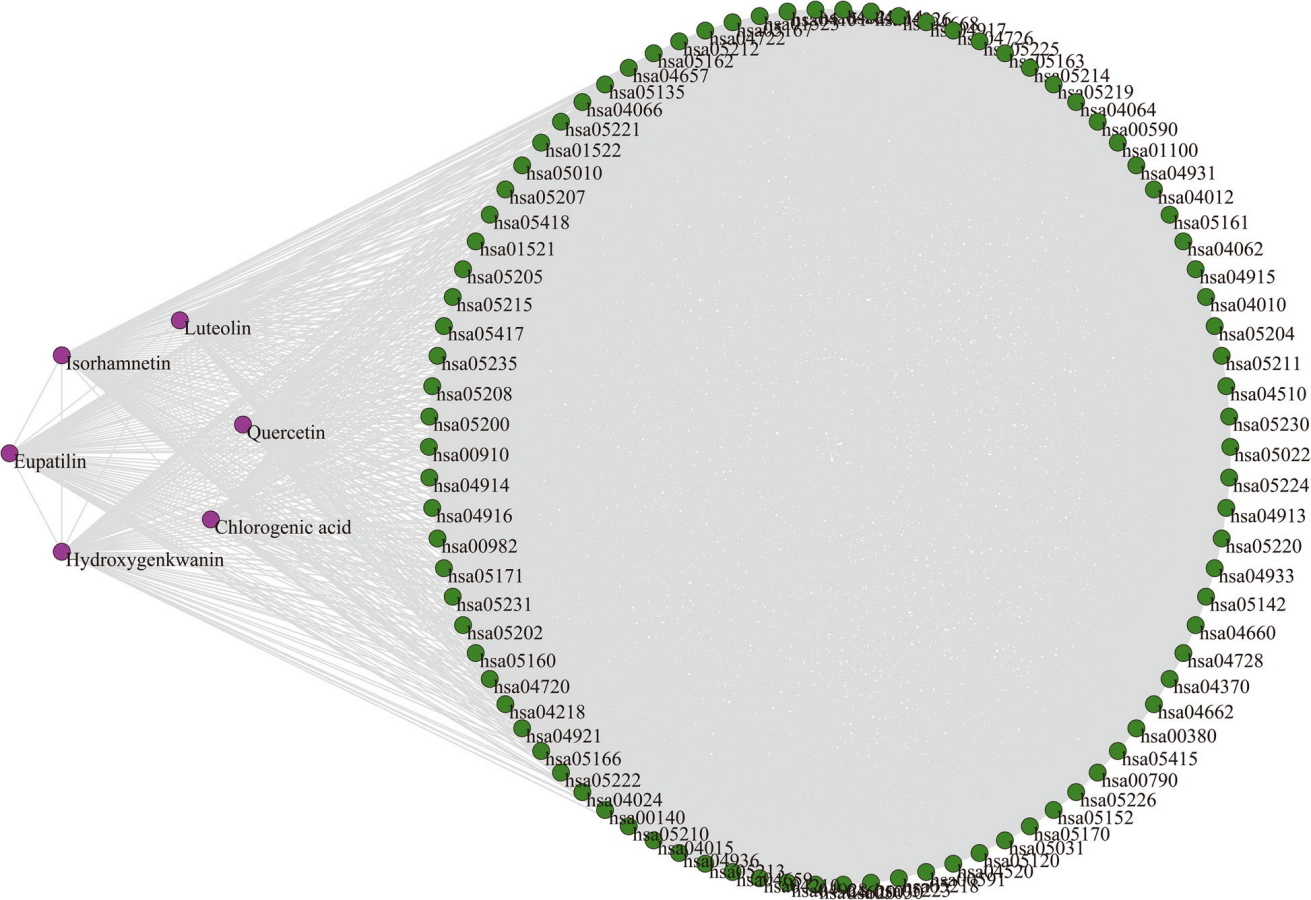
Ingredient-pathway interaction (IPI) network. Purple nodes indicate topo I inhibitors from ASH extract. Green nodes indicate the pathways related to topo I inhibitors from ASH extract. Gray lines indicate their connections

Natural products originated from natural products work through regulation of many key target proteins and metabolic pathways [39]. Multiple complex networks models were applied to investigate the complicated interrelationships for topo I inhibitors from ASH extract. Target searching and pathway analysis were conducted on these ingredients, and that isolated elements were removed. Finally, a total of 160 targets and 90 pathways were reserved for network construction (Additional file 2). An ingredient-pathway interaction (IPI) network was built to describe relationships among the topo I inhibitors and related pathways, based on common target proteins. Figure 4 showed that the network consisted of 96 nodes and 3593 edges. The nodes represented 6 topo I inhibitors and 90 related pathways, and that edges indicated their interactions.

**Fig. 5:**
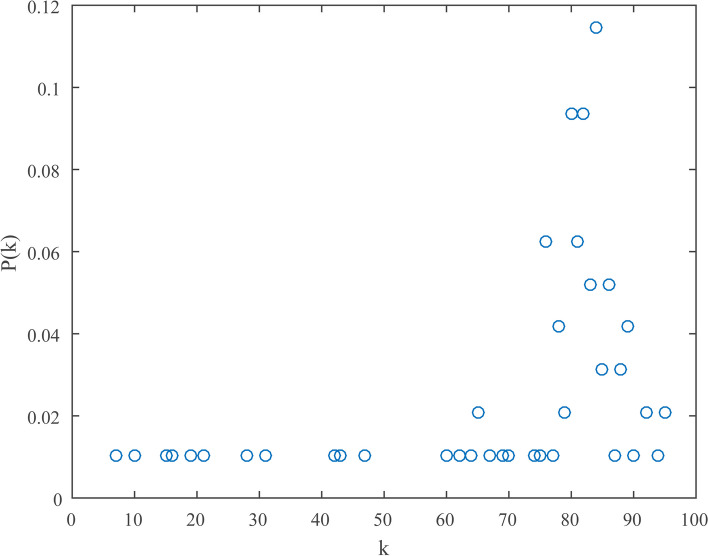
Degree distribution diagram of IPI network. The symbol *k* indicates degree, and that *P(k)* indicates degree distribution

Statistical properties of IPI network were analyzed in detail (Table 2). The results showed large density (0.79) and clustering coefficient (*C* = 0.91), indicating a strong tendency to aggregate. Additionally, an ingredient or pathway node was more likely to connect with the same kind of nodes. A relatively small average path length of the IPI network (*L* = 1.21) suggested that influences on nodes would spread rapidly to rest of the network, and that regulation of critical nodes could affect the whole network. Figure 5 illustrated the probability of a random node with a specific degree *k*. It was found that degrees of most nodes were close to 80, and that average degree of the network was 74.85. The nodes were more likely to be involved in small clusters. These results displayed that a few topo I inhibitors and certain pathways contribute most to the antitumor effect of ASH extract.

**Table 2:** Characteristics of multiple complex networks for topo I inhibitors from *Artemisiae Scopariae* Herba extract

Characteristics	Symbol	Definition ^a^	IPI ^b^	TTI ^c^	IPB ^d^	PPI ^e^
Average degree	<*k*>	$<{k}>=\frac{1}{N}\sum\nolimits_{{i=1}}^{N} {{{k}_i}}$	74.85	5	9.98	68.87
Density			0.79	1	0.89	0.77
Average path length	*L*	$L=\frac{1}{{N(N - 1)}}\mathop \sum \limits_{{i \ne j}} {d_{ij}}$	1.21	1		1.24
Diameter	*D*	$D={\text{max}}\{ {d_{ij}}\}$	2.00	1		3.00
Clustering coefficient	*C*	${C_i}=\tfrac{{2{e_i}}}{{{k_i}({k_i} - 1)}}({k_i} \geqslant 2)$	0.91	1		0.92
Degree centrality	${C}_{d}$	${C_d}=\tfrac{{{k_i}}}{{N - 1}}$	0.79	1		0.77
Betweenness centrality	${C}_{b}$	${C_b}=\sum\limits_{{j(<{k})}}^{N} {\sum\limits_{k}^{N} {\tfrac{{{g_{jk}}(i)}}{{{g_{jk}}}}} }$	0.01	0		0.03
Closeness centrality	${C}_{c}$	${C_c}=\frac{{N - 1}}{{\sum\limits_{{j=1}}^{N} {{d_{ij}}} }}$	0.84	0		0.83

^a^*N* stands for total numbers of nodes in a network. *d*_*ij*_ stands for the shortest path length connecting node *i* and *j*. *g*_*jk*_ stands for the quantity of geodesics from node *j* to *k*

^b^The ingredient-pathway interaction network

^c^The ingredient-ingredient interaction network

^d^The ingredient-pathway bimodal network

^e^The pathway-pathway interaction network

#### Ingredient-ingredient Interaction (TTI) Network

**Fig. 6:**
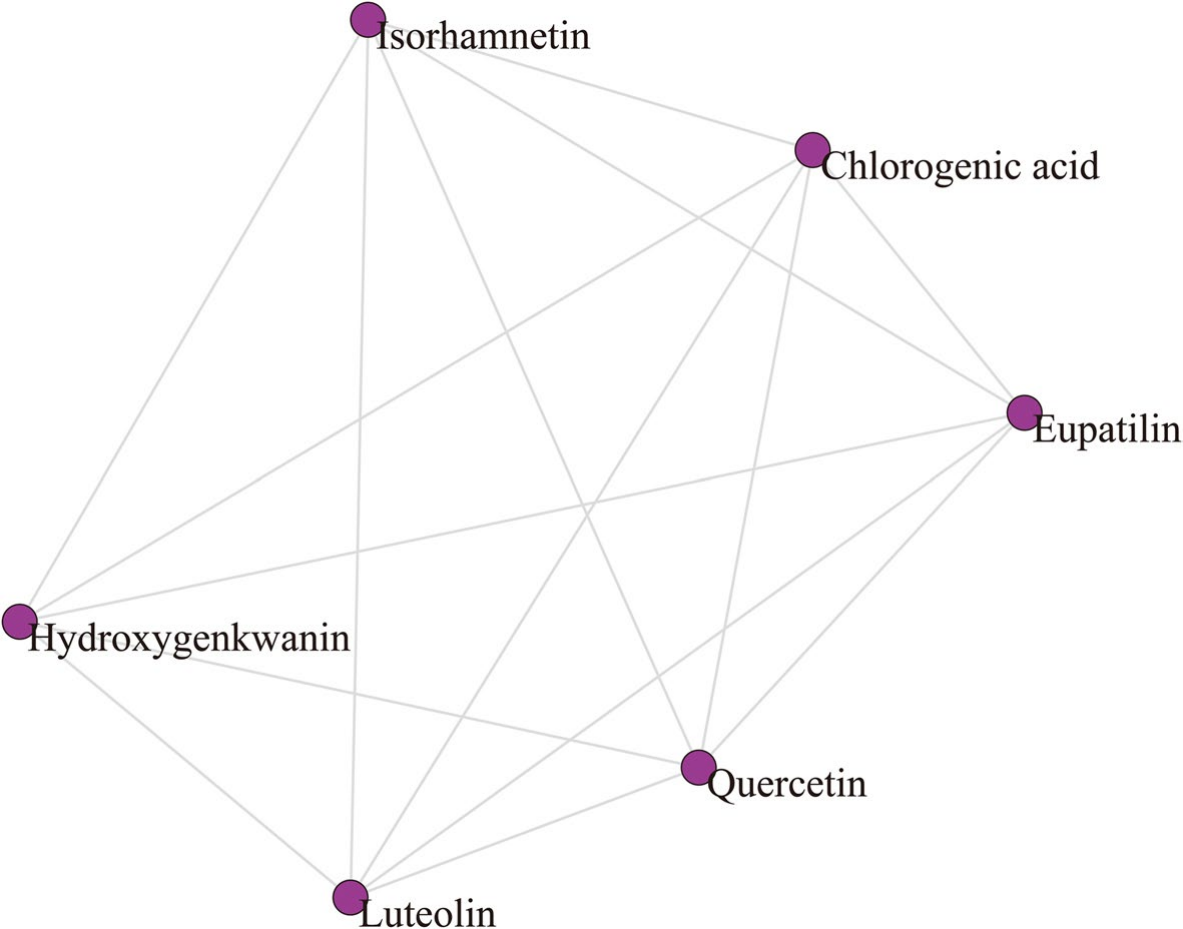
Ingredient-ingredient interaction (TTI) network. Purple nodes indicate topo I inhibitors from ASH extract. Gray lines indicate their internal connections

Three subnetworks were extracted from IPI network for further interpretation, including ingredient-ingredient interaction (TTI) network, ingredient-pathway bimodal (IPB) network, and pathway-pathway interaction (PPI) network. As Fig. 6 shown, TTI network described interactions among the topo I inhibitors from ASH extract, containing 6 nodes and 15 edges. The average path length, density, diameter, and clustering coefficient of TTI network were all very small, with the value of 1 (Table 2). The network was also observed to be a fully connected network, as degrees of all nodes were 5. It demonstrated that any two topo I inhibitors interacted with at least one common target protein. The 6 topo I inhibitors from ASH extract formed a small and highly clustered function module, indicating a potential synergistic effect.

#### Ingredient-pathway Bimodal (IPB) network and main active ingredients

**Fig. 7:**
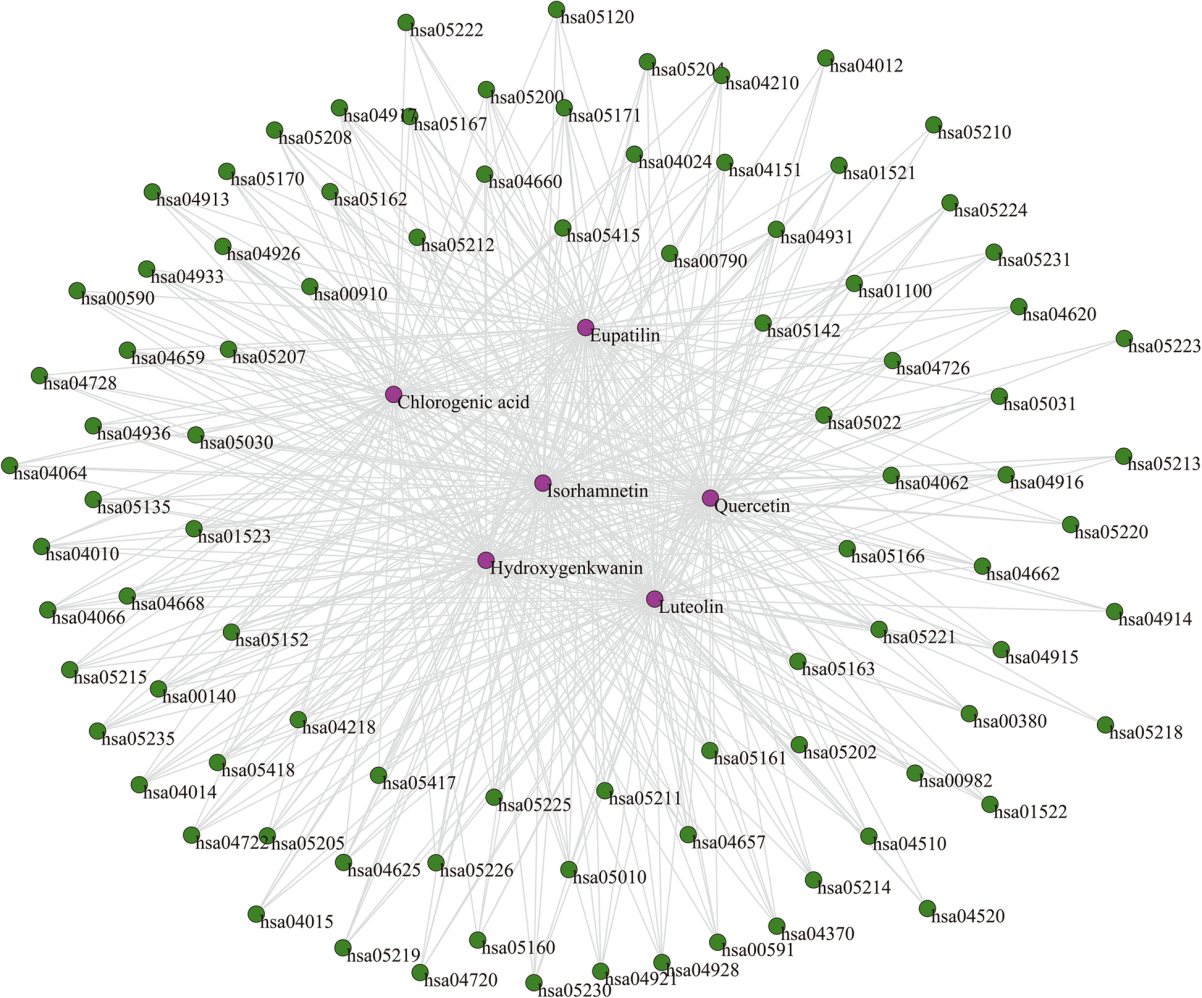
Bipartite graph of the ingredient-pathway bimodal (IPB) network

**Fig. 8:**
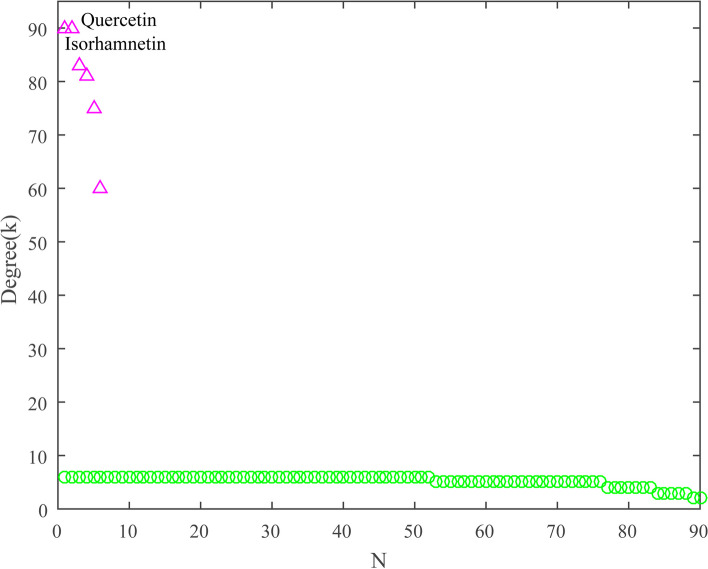
Degrees sorted by descending orders for nodes of IPB network

The ingredient-pathway bimodal (IPB) network was extracted to investigate relationships between topo I inhibitors from ASH extract and pathways related to the same target proteins. This network exhibited as a bipartite graph (Fig. 7), consisted of 96 nodes and 479 edges. Degrees of all nodes were calculated and shown in Fig. 8. Isorhamnetin and quercetin were key nodes of IPB network with the highest degree values (*k* = 90), that linked to all the 90 pathway nodes. As changes in critical positions of the network are always more important than those in marginal or relatively isolated positions [40], the two ingredients were supposed to be main active ingredients. There had been many reports about the antitumor activity of isorhamnetin and quercetin. For instance, isorhamnetin could significantly inhibit the autophagic hypoxia environment of gastric cancer cells, and inhibits cell proliferation [41]. It might trigger paraptosis, and be considered as a new therapeutic approach to oral squamous cell carcinoma [42]. Quercetin showed reliable antitumor effect against hepatoma in vitro and in vivo models [43]. Various nano formulations have been highlighted for quercetin delivery for cancer treatment [44]. These studies confirmed roles of isorhamnetin and quercetin in the antitumor activity.

#### Pathway-Pathway Interaction (PPI) Network and critical pathway

**Fig. 9:**
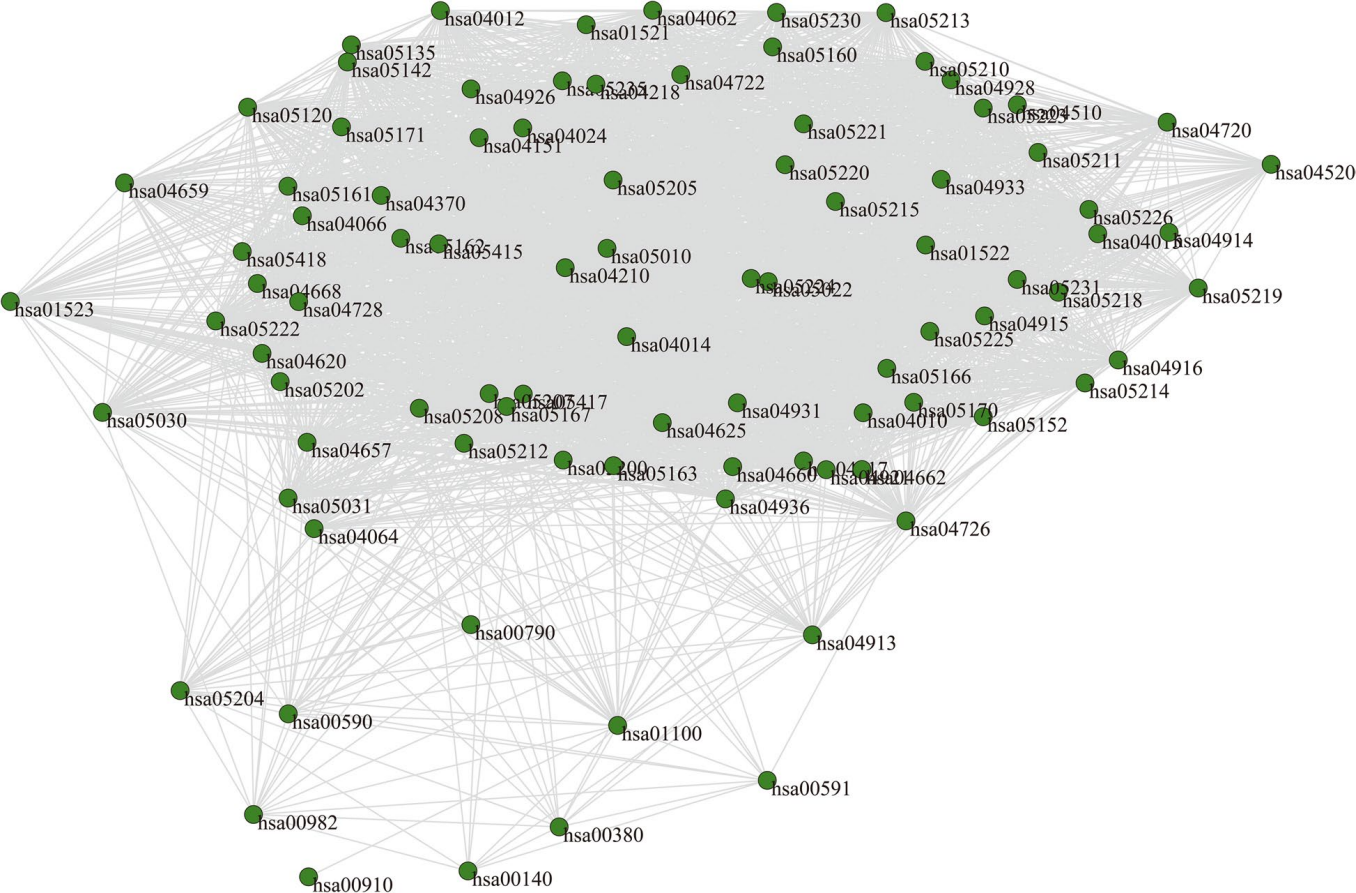
Pathway-pathway interaction (PPI) network. Green nodes indicate the pathways related to topo I inhibitors from ASH extract. Gray lines indicate their internal connections

**Fig. 10:**
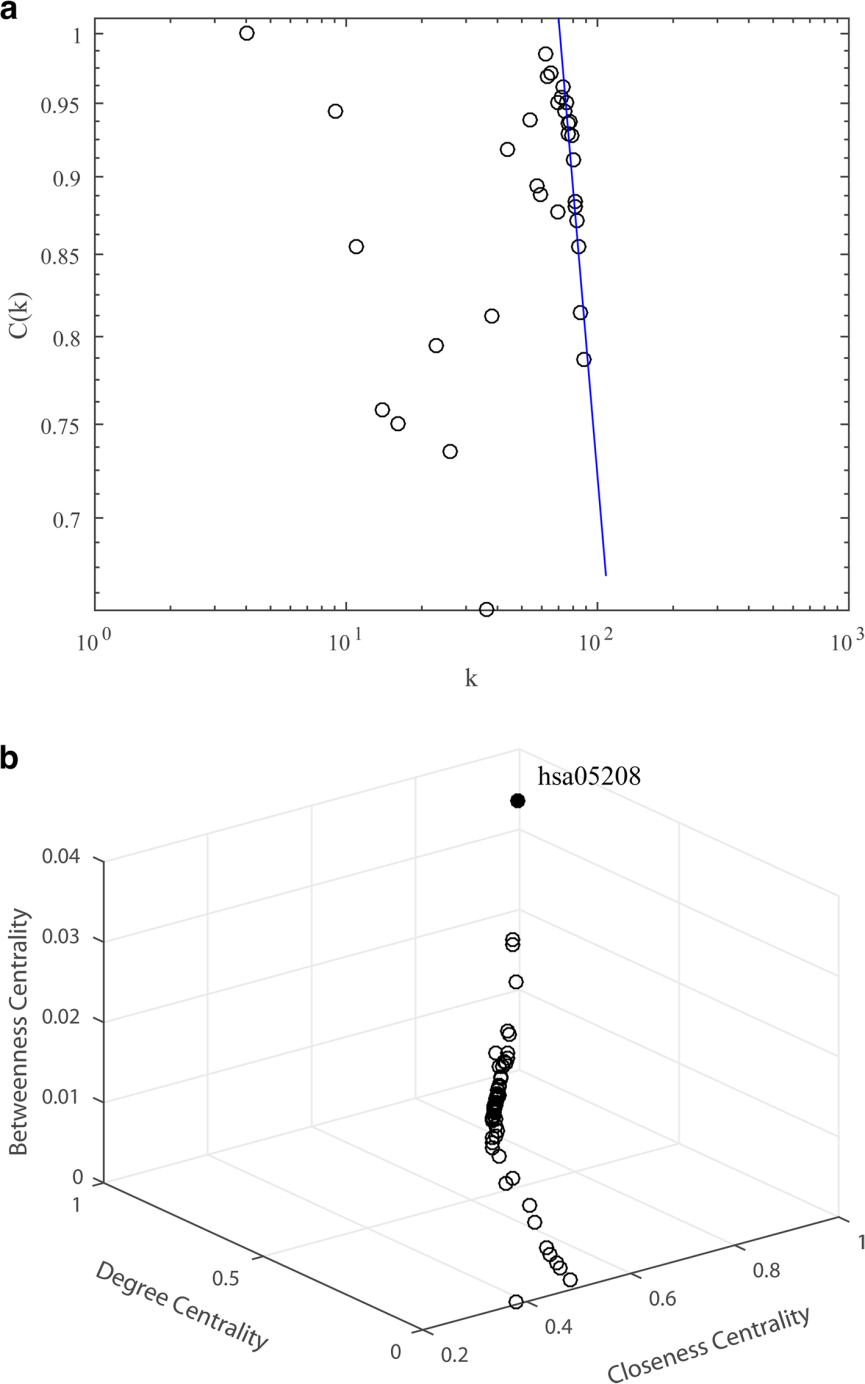
Features of the PPI network. **a**, Average clustering coefficient for nodes in the PPI network. The x-axis indicates degree *k*, and that y-axis indicates the clustering coefficient *C(k)*. **b**, Centrality analysis of nodes in the PPI network, including degree centrality (*C*_*d*_), betweenness centrality (*C*_*b*_) and closeness centrality (*C*_*c*_)

Interrelationships among pathway nodes were also extracted from IPI network, shown as a pathway-pathway interaction (PPI) network (Fig. 9), constituted by 90 nodes and 3099 connections. Figure 10a depicted the correlation between degrees and clustering coefficients of nodes in PPI network. The PPI network exhibited a power-law degree distribution (C_(*k*)_∝*k*^*γ*^, *γ*=-0.95, *k* was great than or equal to 72). Clustering coefficient of this network was relatively high (*C* = 0.92), coupled with a small average path length (*L* = 1.24). It showed a small-world character of the network. This property suggested that many small and loosely linked, hierarchical clusters existed in the PPI network, and formed to be large, less cohesive units. In other words, a few pathway nodes were keys to the overall network. Degree centrality (*C*_*d*_), betweenness centrality (*C*_*b*_), and closeness centrality (*C*_*c*_) were then analyzed to evaluate significances of all the nodes (Fig. 10b). The pathway chemical carcinogenesis-reactive oxygen species (hsa05208) showed much higher centrality indexes (*C*_*d*_ = 0.989, *C*_*b*_ = 0.034, *C*_*c*_ = 0.989). It was supposed to be critical pathway of the antitumor effect of topo I inhibitors from ASH extract.

This pathway displays that a group of carcinogens induce cancer via nongenotoxic mechanisms. The biochemical modes of action for nongenotoxic carcinogens are diverse, one example of which is induction of oxidative stress. Reactive oxygen species (ROS) are also generated due to induction of various cytochrome P450 isoenzymes during detoxification of chemical carcinogens. Increased ROS generation often has been linked to DNA damage that can lead to mutations and may, therefore, play an important role in the initiation and progression of multistage carcinogenesis. Hsa05208 has been considered as key to many kinds of tumors [45–47], such as colon cancer, lung adenocarcinoma, triple-negative breast cancer, and so on. These reports further proved the importance of hsa05208 in the antitumor effect of topo I inhibitors from ASH extract.

### Validation of Topo I inhibition and potential synergistic effect

**Fig. 11:**
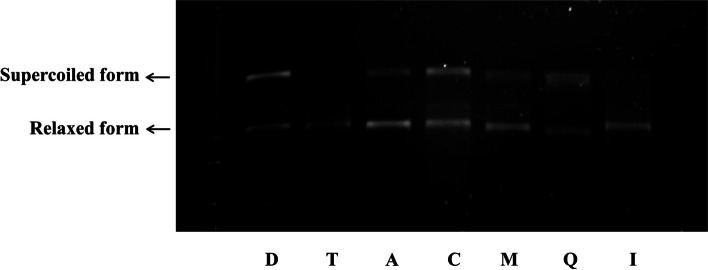
Topo I inhibitory binding assay by agarose gel electrophoresis. Lane “D” represents pure pBR322 DNA (0.175 µg). “T” is the mixture of topo I (0.5 U) and pBR322 DNA (0.175 µg). The same amounts of topo I and DNA were added to all the other lanes. Lanes “A” represents the addition of ASH extract (2.0 µg), and that “C” indicates camptothecin (1.4 µg, positive control). Lanes “M”, “Q”, “I”, represent the addition of the mixture of 1.0 µg quercetin and 1.4 µg isorhamnetin, quercetin (1.0 µg), isorhamnetin (1.4 µg), respectively

A topo I inhibitory binding assay was further performed to validate the results. The inhibition of ASH extract, the main active ingredients isorhamnetin and quercetin, and their mixtures against topo I were evaluated using agarose gel electrophoresis. As shown in Fig. 11, topo I could unspool supercoiled bands of pBR322 into loose DNA bands, and that the positive control camptothecin exhibited a therapeutic effect. Obviously, the addition of ASH extract, and the mixture of isorhamnetin and quercetin with a certain proportion (1.4:1, *w*/*w*) according to the LC-MS results, both showed a similar behavior to camptothecin. Besides, individual addition of quercetin had remarkable inhibitory effect, whereas that of isorhamnetin was relatively weak. These results confirmed the inhibitory effect of ASH extract against topo I, and suggested a potential synergistic effect of isorhamnetin and quercetin, which might be on account of coordination and balance of natural products on topo I-DNA complex [48].

## Discussion

Some previous researches have explored antitumor effect of *Artemisiae Scopariae* Herba. They often focused on of specific constituents or pathways. For example, Prasad et al. reported that cirsiliol suppressed epithelial to mesenchymal transition in B16F10 malignant melanoma cells through alteration of the PI3K/Akt/NF-κB signaling pathway [49]. Hyperoside was found to inhibit lipopolysaccharide-induced inflammatory responses in microglial cells via p38 and NFκB pathways [50]. A comprehensive and detailed analysis of bioactive ingredients in ASH extract is still needed. In this study, interdisciplinary methods were integrated as an extensive approach, including bioaffinity ultrafiltration-UFLC-ESI-Q/TOF-MS/MS, *in silico* docking and multiple complex networks. First, the application of ultrafiltration LC-MS could facilitate the separation of ligands binding to target proteins from natural products [51]. On the other hand, the specific binding sites of protein residues or functional groups of natural products were clarified by *in silico* docking, which determine the topo I inhibitory activity [18]. Finally, complex network methodology was used to explore the underlying mechanisms, with a significant potential for extracting key biological information from large amounts of data [52]. Combination of these methods enabled to get an overall understanding of topo I inhibitors in ASH extract from a system perspective, containing main active ingredients, target proteins and critical pathways. However, further studies on cell and animal models are needed to verify these results.

Currently, natural topo I inhibitors used in clinical treatment of tumors are mainly originated from camptothecin and its derivatives, including topotecan against ovarian and cervical cancers, irinotecan against colorectal and pancreatic cancers, as well as belotecan, widely used against small cell-lung cancer [18]. The present results of ASH could provide another potential alternative to the development of clinical anticancer drugs. Besides, drug combinations of topo I inhibitors and other biologically active compounds is the other key issue [17]. It was reported that camptothecin complexed with the polymer doxorubicin had synergistic antitumor activity, and increased the drug accumulation in tumors [53]. A similar synergistic effect of isorhamnetin and quercetin was also observed in this study. Nevertheless, more pharmacological researches are essential to solving the puzzle. We hope that further evidence-based clinical studies focused on ASH and ASH-derived bioactive ingredients could open new avenues to tumor therapy.

## Conclusion

This research presents one of the first multi-disciplinary surveys of topoisomerase I inhibitors from medicinal plants. The *Artemisiae Scopariae* Herba extract exhibited toxicogenetic and antiproliferative activities on A549 cells. A series of 34 ingredients were identified, and 6 of them were screened as potential topo I inhibitors. Their fragment patterns in ESI source were analyzed in detail. Molecular docking showed that the formation of hydrogen bond and π-π stacking contributed most to the binding of topo I and the inhibitors. The inhibitors, related targets and pathways were analyzed in multiple complex networks model, which showed a power-law degree distribution and small-world property. Network analysis revealed that isorhamnetin and quercetin were main active ingredients, and that chemical carcinogenesis-reactive oxygen species (hsa05208) was the critical pathway. In addition, the ASH extract exhibited therapeutic effect on the conversion of supercoiled DNA to relaxed forms, and a potential synergistic effect of isorhamnetin and quercetin was also observed, which would be studied in our next study. The results improved current understanding of *Artemisiae Scopariae* Herba on the treatment of tumor. The combination of bioaffinity ultrafiltration-UFLC-ESI-Q/TOF-MS/MS, *in silico* docking and multiple complex networks provided a new strategy for the study of bioactive constituents in medicinal plants.

### Supplementary Information

**Additional file 1.** Results of molecular docking for topo I and potential inhibitors from *Artemisiae Scopariae* Herba extract

**Additional file 2.** Collection of target proteins and pathways for topoisomerase I Inhibitors from ASH extract

## Data Availability

Datasets supporting the results of this article have been included in the additional files.

## Abbreviations

ASH: *Artemisiae Scopariae* Herba
topo I: topoisomerase I
UFLC: Ultra fast performance liquid chromatography
ESI: Electron spray ionization
Q-TOF: Quadrupole time-of-flight
MS: Mass spectrometer
IPI: Ingredient-pathway interaction
TTI: Ingredient-ingredient interaction
IPB: Ingredient-pathway bimodal
PPI: Pathway-pathway interaction
DNA: Deoxyribonucleic acid
IL-6: Interleukin 6
STAT3: Signal transducer and activator of transcription 3
SRB: Sulphorhodamine B
FBS: Fetal bovine serum
SEA: Similarity ensemble approach
ROS: Reactive oxygen species
hsa05208: Chemical carcinogenesis-reactive oxygen species
PDB: Protein data bank
HCC: Hepatocellular carcinoma
VEGFA: Vascular endothelial growth factor A
TP53: Tumor Protein P53, IL-6:interleukin-6
TNF: Tumor necrosis factor
MARK1: Microtubule affinity regulating kinase 1
DMEM: Dulbecco’s modified eagle medium
FBS: Fetal bovine serum
DMSO: Dimethyl sulfoxide
