# Supplementary material for: Combination of bioaffinity ultrafiltration-UFLC-ESI-Q/TOF-MS/MS, in silico docking and multiple complex networks to explore antitumor mechanism of topoisomerase I inhibitors from Artemisiae Scopariae Herba
Source: BMC Complement Med Ther. 2023 Sep 12;23:317. doi: 10.1186/s12906-023-04146-x (PMC10496380; doi:10.1186/s12906-023-04146-x)
Supplement: Supplementary file 1 — Additional file 1. Results of molecular docking for topo I and potential inhibitors from Artemisiae Scopariae Herba extract [file 12906_2023_4146_MOESM1_ESM.docx]

**Additional file 1** Results of molecular docking for topo I and potential inhibitors from *Artemisiae Scopariae* Herba extract

| **Topo I inhibitors** | **-CDOCKER interaction energy (kcal·mol^-1^)** |
| --- | --- |
| Chlorogenic acid | 53.99 |
| Quercetin | 75.75 |
| Luteolin | 68.19 |
| Eupatilin | 55.89 |
| Hydroxygenkwanin | 53.73 |
| Isorhamnetin | 74.61 |
| Camptothecin ^a^ | 37.76 |

^a^ Camptothecin was used as control.
